# Enhancing evidence use in public health nutrition policymaking: theoretical insights from a New Zealand case study

**DOI:** 10.1186/s12961-016-0154-8

**Published:** 2016-11-25

**Authors:** P. Field, R. Gauld, M. Lawrence

**Affiliations:** 1 Department Human Nutrition, University of Otago, P.O. Box 56, Dunedin, 9054 New Zealand; 2 Department of Preventive and Social Medicine, Center for Health Systems, University of Otago, P.O. Box 56, Dunedin, 9054 New Zealand; 3 Institute for Physical Activity and Nutrition, Deakin University, 221 Burwood Highway, Burwood, Victoria 3125 Australia

**Keywords:** Evidence use, Policy, Advocacy, Public health nutrition

## Abstract

**Background:**

Enhancing the use of evidence in policymaking is critical to addressing the global burden of nutrition-related disease. Whilst the public health nutrition community has embraced evidence-informed policymaking, their approach of defining relevant evidence and evaluating policy has not brought about major shifts in policymaking. This article uses a public health nutrition case study to refine a novel theory-informed framework for enhancing the use of evidence in government public health nutrition policymaking. Our aim is to contribute insights from evidence-informed policy to the emerging paradigm in public health nutrition policymaking.

**Methods:**

An enquiry framework informed by three groups of theories underpinning evidence-informed policy was used to explore the role of socially mediated processes on the use of evidence. A public health nutrition case study on food marketing to New Zealand children was conducted to refine the framework. Interview data collected from 54 individuals representing four key policy stakeholder groups, policymakers, academics, and food industry and non-government organisations were analysed using deductive and inductive thematic analysis. To enhance theoretical robustness, an alternative hypothesis of political explanations for evidence use was explored alongside the enquiry framework.

**Results:**

We found the prevailing political climate influenced the impact of advocacy for evidence inclusive processes at the meta-policy and policymaking process levels and in policy community relationships. Low levels of awareness of the impact of these processes on evidence use and uncoordinated advocacy resulted in the perpetuation of ad hoc policymaking. These findings informed refinements to the enquiry framework.

**Conclusion:**

Our study highlights the role advocates can play in shifting government public health nutrition policymaking systems towards enhanced use of evidence. Our Advocacy for Evidence Use framework argues for a three-channel approach to advocacy for using evidence in the public interest. The framework provides a means for building a constituency for evidence use in public health nutrition and adds understanding about advocacy to the field of evidence-informed policy. Future research should examine the impact of coordinated advocacy on public health nutrition policymaking systems.

**Electronic supplementary material:**

The online version of this article (doi:10.1186/s12961-016-0154-8) contains supplementary material, which is available to authorized users.

## Background

Dietary risk factors are now the leading contributors to the global burden of disease [1]. In particular, suboptimal nutrition is driving many aspects of global patterns of disease. As rates of overweight and obesity continue to increase unevenly around the world, attention is turning to government systems for the development of remedial policies [2]. Patterns in New Zealand reflect these trends, with 31% of adults being obese and Maori and Pacific adults having higher rates of obesity, of 47% and 66%, respectively [3]. For decades, leading nutritionists have been calling for a new paradigm in nutrition policymaking where evidence from a variety of sources is systematically considered [4–6]. The 2015 *Lancet* series on obesity argues for ‘new thinking’ by addressing issues at both the individual and environmental level and pursuing policy-based approaches to changing the food environment [7–10]. However, for these science-based arguments to gain traction this evidence-policy interface requires closer examination [11]. We define evidence broadly including quantitative, qualitative and synthesised evidence produced by a range of methods.

Government policymakers are confronted with a number of challenges in protecting and promoting public health nutrition (PHN). The problems for which they need to formulate policy are often complex, substantial and contested. Although evidence-informed policymaking is promoted as the ideal, it rarely takes place as a rational and linear process. Instead, advocacy has a significant influence on how and why the decision making process plays out [12, 13]. Here, we define advocacy as working proactively to change upstream environmental factors, in particular the policies, regulations and institutional practices which influence the personal health choices of large population groups [14]. Effective policy advocacy involves a range of interpersonal and organisational level strategies orchestrated by skilled individuals whose primary activities are building advocacy coalitions and engaging with decision makers in a bottom up manner [15]. In this regard, as we have argued elsewhere [16], there are three areas where advocacy can explain when evidence is used in the policymaking process.

Advocacy by influential policy actors with the aim of increasing evidence use has not been systematically examined beyond three lines of enquiry: Nutley et al.’s [17] application of diffusion of innovation frameworks to the idea of evidence use; Sabatier et al.’s [18] Advocacy Coalition Framework; and Greenhalgh et al.’s [19] systematic review of diffusion of innovation in health service organisations. The aim of this article is to use empirical and theory-informed conceptual thinking to build a framework for using a variety of evidence in PHN policymaking. Our earlier framework on the role of advocates in evidence-informed policymaking [16] is tested and refined in a PHN policy case study in the New Zealand environment. PHN policymaking in New Zealand provides a rich case for a study on advocacy for evidence use. It is a discrete policy area with a number of high stakes policy issues with scientific evidence bases and clearly identifiable actors, yet from 2008 to 2014, the Government had a position of minimal action. The revised advocacy framework provides additional features to the Institute of Medicine, World Obesity, and WHO models for advancing evidence use in PHN policymaking [20–22].

### Enquiry framework

The Role of Advocates for Using Evidence framework argues that advocates are able to shift (1) meta-policy arrangements, (2) PHN policy community relationships, and (3) policymaking processes to enhance the use of evidence [16]. These three aspects of PHN policymaking amenable to advocacy emerged from our mining of theoretical frameworks addressing the types of evidence used in public health policymaking, the influence of power and interests on evidence use, and social aspects of policy processes. We focused on identifying socially mediated policymaking structures and processes that exert considerable influence on how, when and why evidence is used. The enquiry framework portrays these three mutually reinforcing aspects as policymaking components harnessing the wider context of policy-relevant knowledge to produce evidence-informed policy.

#### Meta-policy

Meta-policy, the highly influential written and unwritten rules about policymaking, effectively determine the use of evidence in policy processes. These ‘rules’ govern who is included and excluded, time frames for decision making and the extent and status of external input [23, 24]. Drawing on a social interaction theory, diffusion of innovation, this component proposes that opinion leader advocates’ role is to work through existing PHN advocacy coalitions to direct advocacy towards meta-policy level influences on evidence use [25].

#### Sustained relationships

This component proposes that sustained relationships between broadly based members of a policy community enable effective advocacy for evidence use. Here, the role of skilled individuals is to unite networks across PHN policy communities and across policy issues by diffusing the novel ‘idea’ that enduring relationships are critical for effective advocacy. Advocacy is for policy processes that promote the public interest through consideration of broadly based evidence.

#### Deliberative processes

Building on Flitcroft’s argument that deliberative policymaking processes have the potential to reframe policy dilemmas and resolve policy controversies, this component proposes that the role of advocates is to pursue inclusive structures and systematic processes [24]. Their goal is Lomas et al.’s [26] well known deliberative policymaking process model, particularly consultation with all parties affected by an outcome, inclusion of a fair representation of scientists and other stakeholders, high-quality syntheses of the scientific evidence and skilful chairing.

## Methods

### Case study design

This study sought to produce a robust explanatory framework through a real world policy case study. A deductive approach was used to refine an existing conceptual framework developed from a synthesis of literature relevant to evidence-informed policy. Policy case study interview data explored PHN policy actors’ views on the ability of the framework to explain advocacy for using evidence. Concurrently, a grounded theory approach was used to explore emerging explanatory themes. A rival hypothesis, political explanations for evidence use, was explored using the same approach. Food marketing to New Zealand children was chosen as an exemplary single case study. This policy area provided rich case study data as a well-established evidence base existed, a range of identifiable policy actors were involved, there was controversial meta-policy and the New Zealand Government had a clear policy position, see Additional file 1 Case Study background. The University of Otago Human Ethics Committee granted ethical approval for the qualitative interviews.

### Data collection

In-depth conversational interviews explored the role of advocates for using evidence in PHN policy on food marketing to children. Members of the New Zealand PHN policy community with an interest in food marketing to children were relatively easily identifiable by the snowballing method. The first interviewees were identified through having a senior position in an organisation affected by food marketing to children policy and by being known for having or being able to influence policy processes. Individuals without organisational affiliations or with no profile on food marketing to children policy were excluded. Over 9 consecutive weeks (July–August 2012), 54 in-depth conversational interviews, lasting 30–40 minutes, were conducted either by telephone or face-to-face. Four distinct groups emerged within this policy community: policymakers, the political and bureaucratic decision-makers; policy active academics, the sources of scientific evidence; PHN non-governmental organisation (NGO) groups, the organised civil society groups with interests in policy and evidence; and the food industry, the companies and coalitions with a high stakes involvement in policy processes. All interviewees were asked open-ended questions on their experiences of advocacy for evidence use. Questions were structured around the three components of the enquiry framework and how politics influenced the use of evidence. Additional file 2 contains the interview guide.

The interview sample comprised 16 policymakers, 13 middle to senior level food industry employees, 13 managers in non-government organisations, and 12 research-active academics interested in policy. As far as possible, interviews were conducted in blocks by category group. Interviews were transcribed within a short time frame of the interview using voice recognition software.^1^ To ensure the sample size was large enough to produce sufficient rich and comprehensive data for the intended analyses, interviews within each category were continued until convincing redundancy of information, i.e. data saturation, had been achieved.

### Data analysis

Respondents were assigned a group identifier to identify congruent and divergent views by group: policymakers, academics, NGOs, and food industry. Group membership appeared stable at the time of the interviews, with no interviewee purporting to be a member of more than one group. To ensure anonymity, interviewees were assigned a unique code and number, e.g. PM 1, was an individual policymaker.

Thematic analysis was performed on the fully transcribed interview data using the four category themes generated by the enquiry framework: meta-policy, sustained relationships, deliberative processes and the alternative explanation, political influences. Initial coding allocated comments as supporting or disagreeing with the enquiry framework components or the alternative explanation, or as a potential emerging theme. There were iterative rounds of data review to confirm the allocation of text comments to a code. Subthemes developed based on frequency of coded responses and were merged following a critical review of code allocation and examination of common conceptual links. Major category theme and subtheme descriptions were then refined. PF undertook the initial coding. This was checked by RG and ML.

## Results

The results are organised around the role of advocates in the three components of the enquiry framework: meta-policy, sustained relationships and deliberative policy processes. The role of politics is also explored as an alternative explanation. Whilst food marketing to children was the entry point for the interviews, it was apparent early in each interview that issues in the wider PHN policy environment explained the current situation on food marketing to children. Consequently, data was obtained on both food marketing to children and broader PHN issues.

### Meta-policy

In New Zealand, institutions inside and outside government have a government mandate to develop and implement nutrition-related policy. The key institutions are two government departments, the Ministry of Health (MoH) and the Ministry for Primary Industries, MoH Public-Private Partnerships (PPPs) with a major NGO and with the Food Industry, and the non-regulated industry-led Advertising Standards Authority.

In government-funded organisations, formal meta-policy structures and processes exerted a major influence on the impact of any advocacy for using evidence. For the MoH, the dis-establishment in 2009 of their Nutrition Advisory Committee represented a significant structural barrier to further evidence-informed policy development. Many aspects of PHN policy advocacy were affected by the removal of this key committee known for rigorous consideration of scientific evidence. The Nutrition Advisory Committee was not alone, as the centre right Government in their first term of office (elected in 2008) disbanded a number of expert advisory committees. A senior policymaker observed:

“*…because at the time the ministry had a vast number of advisory committees for just about any everything, it was questionable how much value they added*.” Policymaker 6

The same Minister’s (2008–2014) well-known hands-on approach and aversion to discussing public health issues became a barrier to advocacy for creating alternative structures for providing evidence-informed policy advice. Formal channels for science-based evidence-informed policy advice were further compromised by an internal restructuring of government departments, where technical nutrition experts were replaced by politically pliable non-specialist policy analysts.

These meta-policy changes, while not impacting on the type of evidence most groups were advocating, did impact on channels used by academics and smaller NGOs. The food industry and larger NGOs continued to use their own well-oiled direct advocacy channels. With the loss of their only structural channel for scientific evidence to reach policymakers, smaller NGOs and academics increased their use of ad hoc informal processes. Larger NGOs continued proactive advocacy directly to policymakers for their more broadly based scientific and contextual evidence to be considered. Similarly, the food industry continued to use direct advocacy, although with an increased emphasis on scientific evidence alongside their market-based data.

Members of the food industry were also proactive with informal advocacy to shift meta-policy towards arrangements where industry had a larger role in policy development. Most academics and smaller NGOs were more reactive responding to issues as they arose, with little if any awareness of the influence of meta-policy. As newer collaborative meta-policy arrangements, PPPs were increasingly common across government and bureaucrats believed they had political support to develop partnerships with the food industry. However, the only NGO working in a MoH-funded PPP perceived a power imbalance that compromised their independence.

The role of civil society groups as the ‘voice of evidence’ in formal and informal policy discussions emerged as an important issue for NGO and academic interviewees. In the prevailing political climate, civil society voices were seen as having more influence than scientific experts, particularly when a constituency was built for public health action. Some NGOs deliberately engaged in ‘sideways advocacy’ by packaging scientific evidence to enable community group voices to speak the evidence.

### Sustained relationships

**Fig. 1:**
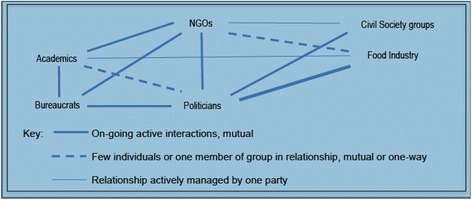
Mutuality in linkages between major players in the New Zealand public health nutrition community.

As informal policy processes dominated nutrition policymaking, most social interactions across the policy community were based on informal relationships. Despite only a few formal committees facilitating relationships between sectors, informal mutually beneficial relationships flourished. The relationship patterns captured below (Fig. 1) portray how differing levels of mutuality influenced levels of interaction between key groups.

Advocacy for formal ongoing relationships between major groups in the policy community was distinctly limited. Formal relationships existed only where structures enabled relationships between individuals from different sectors. Only the two major nutrition NGOs had academics as long-standing members of advisory committees. These relationships were considered to have high levels of mutuality. One business industry NGO also maintained a number of advisory groups with a broad membership, and a notable absence of academics.

Senior health bureaucrats reported enjoying long-standing, informal relationships with senior academics where either party would telephone the other for advice or an alternative perspective on an issue. These relationships often began as formal relationships and became sustained by mutuality arising from trust, respect and exchange of information. Most Members of Parliament (MPs) also had their own informal relationships with trusted academics. However, these relationships were short lived compared to bureaucrats’ relationships. More politically attuned NGOs also initiated and maintained relationships with elected MPs and senior bureaucrats.

For the food industry, initiating and maintaining informal relationships with politicians and bureaucrats was a priority activity. Food industry members frequently coordinated their engagement with government through representative groups, including a powerful coalition, the Food Industry Group, established in 2003. In comparison, nutrition NGOs could not sustain coordinated advocacy. Competition between academics based on University affiliations combined with the disjointed NGO sector created difficulties for bureaucrats seeking external public health input on policy issues. A senior bureaucrat commented:

“*Who is public health, is it the Public Health Association, or is it some key academics, who is it?… Industry is easier because there are key bodies.*” Policymaker 3

### Deliberative policy process

Deliberative policymaking processes were virtually non-existent, as most policy processes used selected evidence in selected ways. For many bureaucrats, political interference and the lack of systematic internal processes for reviewing public health evidence meant they were only involved in advising on the efficacy of implementation options and had no opportunity to advocate for alternative processes.

Several major food companies initiated a type of deliberative policy process in providing scientific and other evidence directly to policymakers, believing this added credibility to industry submissions and interactions with MPs. One food industry representative understood their material provided bureaucrats with evidence that was not available from other sources:

“*They would take all the science they could get, they know we have the science at our fingertips and will ask for it, not just science but evidence in general, sales figures.*” Food industry member 4

NGOs, on the other hand, provided scientific evidence to government through formal structures, committees and consultation rounds. Experienced advocates in all groups were keenly aware that their scientific evidence-based advocacy had minimal impact compared to other policy inputs.

As most NGOs were dependent on government funding they pursued a politically pragmatic and conservative approach to PHN advocacy. Senior NGO interviewees attributed the high level of political involvement in PHN policy processes to New Zealand’s Mixed Member Proportional electoral system. A succession of coalition governments with small majorities over the last decade had forced MPs to be responsive to their electorates and party ideology. A NGO representative believed this political fragility enhanced the influence of civil society groups.

Advocacy by some policy active academics was hindered by fear of being ‘labelled’ by politicians and bureaucrats when their views differed from Government ideology. These academics believed politicians and senior bureaucrats’ perceptions of their personal political views determined whether their advice was sought. Some expressed concern around the impact of being labelled on future competitive government research funding applications.

## Discussion

A complex relationship between evidence and policymaking was evident in this investigation of advocates’ role in three channels that influence evidence use in PHN policymaking. Much of this complexity arose from interdependencies between the channels working to facilitate or hinder the use of evidence.

Gibson’s argument for the role meta-policy plays in evidence use highlights the need for evidence use advocates to be aware of meta-policy [23]. Our results indicate a low-level of awareness of formal and informal meta-policy helps explain the limited advocacy for enhanced use of evidence. Head recognises the need for political incentives to promote organisational climates and cultures supportive of evidence-informed decision-making processes [27]. However, it appears that similar incentives are needed at the meta-policy level to shift organisations towards evidence-informed policymaking. In this study, the lack of powerful political incentives for alternative meta-policy may be attributed to three coinciding factors. A weak policy community being largely unaware of meta-policy, a hands on Minister of Health and influential senior bureaucrats maintaining the status quo by continually framing advice to be acceptable to their Ministers.

A major consequence of low profile meta-policy is an absence of transparency. When industry groups advocate for a change in meta-policy arrangements this is seen by groups with public health values as an exercise of asymmetrical power by another group with conflicting values. In the field of evidence-informed policy, the principle of transparency is frequently applied to high quality, accessible evidence and policy processes yet rarely to meta-policy guiding how evidence is used [27–29]. This relationship between low profile and transparency creates problems for the wider policy community as it prevents consideration of the harm and benefit consequences of the meta-policy level ‘rules’ for using evidence.

In relation to the second channel, that of sustained relationships, the findings extend the work of Flitcroft et al. [24], Hanney and Gonzalez-Block [30], and Lavis et al. [31], who promote the role of organisational structures in supporting dialogue on specific policy issues. Our work indicates that, when these structures exist over time and across policy issues, they are important for sustaining relationships across a policy community. For public health groups an influential government-led committee was pivotal for sustaining relationships when it existed, whereas for food industry members their advocacy coalition was a uniting structure.

Along with transient formal structures, the pattern of long-standing networks across the policy community can help explain uncoordinated and conflicting advocacy activities. In the case study, food industry groups exhibited Sabatier’s characteristics of advocacy coalitions [18]. Groups were united by high-level interests around retention of industry self-regulation, which in turn directed advocacy activities and ultimately determined the longevity of a key coalition.

In contrast, NGO and academic groups failed to coordinate their activities. These groups functioned as small clusters of tightly linked individuals, more typical of social networks who do not have collective policy goals [32]. Organised advocacy was constrained by limited resources relative to larger well-resourced food industry groups. Relationships between individuals were strong, being sustained over long periods of time and across policy issues by mutual trust and resource dependence around the exchange of knowledge. However, NGO and academic coalitions could not be sustained since members disagreed on middle-level values around priorities for nutrition policy. Interestingly, these disagreements overrode agreement on high-level beliefs around the primacy of scientific evidence and the crucial role of nutrition in promoting health.

Sabatier argues that beliefs at all levels influence how coalition members respond to new information [18]. Our analysis suggests medium-level beliefs influence the establishment and longevity of advocacy coalitions. Because belief disagreements occurred in weak policy community groups, together, these two social influence factors explain the un-coordinated advocacy. For evidence use advocacy, this analysis highlights the need to align both medium and high-level beliefs of policy community subgroups.

Interestingly, although most members of outside government advisory committees fulfil Rogers’ criteria for opinion leaders they did not attempt to influence wider policymaking systems [25]. This indicates a second area where low levels of awareness hinder advocacy. Cognizance of how on-going relationships facilitate the conceptual use of evidence is needed to address so-called ‘wicked problems’ [33] in PHN policy, where causes and solutions are highly contested. In Rogers’ diffusion of innovation model, persuading opinion leaders of the relative advantage of an innovation is the first step in the adoption of new ideas. This suggests that persuading opinion leaders of the merits of establishing structures that support on-going relationships is a priority issue for the policy community.

In examining deliberative processes, the third channel, two government actions undermined policymakers’ ability to systematically use broadly based evidence. Disestablishing the Nutrition Advisory Committee and side-lining MoH technical nutrition experts prepared the ground for more politically driven policymaking processes. The resulting reduction in structural capacity for addressing complex issues through on-going conversations between evidence producers and users helps explain the rise in ad hoc policy development [34].

Compared to Lomas’ model for deliberative policymaking where skilled, transparent synthesis of evidence includes stakeholders’ values, informal politically driven processes appeared to permit only highly selected evidence to inform policy development [26]. In the absence of skilfully chaired deliberations by balanced stakeholder groups including scientists the outcome could be considered ‘ill-informed’ policy, in comparison to Lavis et al.’s [35] construct of ‘well-informed’ policy.

Furthermore, without formal government-led policymaking structures and processes or rules for engagement, food industry groups have a rationale for building relationships with senior bureaucrats and politicians. As policymakers’ source of readily accessible ‘scientific advice’, industry representatives exert considerable influence over policy processes. Although the food industry meet Lomas’ criteria of a stakeholder affected by the outcome of a policy decision, their role in collaborative processes is not explicitly addressed in the deliberative processes literature [26]. This highlights a limitation of existing models for deliberative health policy processes: the unstated assumption that all groups involved share a public good interest in promoting health_._

### Politics – alternative hypothesis

Diverse explanations exist for the political influences on what, how, when and where evidence is used in policy processes. These range from the exercise of structural power and the influence of policymaking systems through to changing models of governance. The political nature of PHN policymaking has been attributed to competition between the values, beliefs and power bases of diverse stakeholders [6, 13, 36, 37]. Political explanations for evidence use suggest that the way an issue is framed influences what counts as evidence and how it is managed by the political system [27, 38].

In framing obesity as an individual issue, structurally powerful industry groups aligned their position with prevailing political ideology. Jenkin et al. [39] argues this policy frame has determined what counts as policy-relevant evidence and explains the New Zealand Government’s inaction on PHN policy.

In addition, three policy systems level factors offer further political explanations for the role of advocates in influencing the use of evidence. Firstly, although New Zealand civil society groups are politically influential, their low profile on PHN issues prevented community groups from being a voice for evidence. Secondly, the lack of coordinated advocacy between NGO and academic members of the PHN community is partly attributable to internal politics. While this situation may arise from local conditions, Gibson observes similar patterns across the health sector. As health evidence is complex, rational evaluations are only possible when values are considered concurrently [23]. As PHN policymaking processes prevented values and evidence being considered transparently, differences between these groups were exacerbated. Thirdly, the Government’s practice of ‘branding’ individuals and dismissing senior expert advisors whose views did not align with their ideology generated wariness among most academics about engaging in political advocacy. This finding supports Lin’s view of researchers and policymakers operating in competing rationalities, which do not predispose either group to engage easily with the other [40]. Together, these system-level factors created a situation where the wider public health community exerted no political pressure on policymaking systems.

Emerging shifts in PHN governance add another layer of complexity to the systematic and transparent use of evidence. New governance arrangements in New Zealand and Australia are similar to those in other sovereign states [41–43]. Ryan’s [41] proposition that collaboration, partnership and co-production underpin these approaches to public management fails to address issues of transparency and accountability for evidence use.

Explanations for the food industry’s high level of political influence often highlight the considerable resources devoted to a range of relationship management activities [44–47]. Lewis [32] draws attention to an aspect of this influence where Australian health interest groups provide knowledge not easily available to the bureaucracy. In this study, the food industry employed broad and targeted influence strategies indicating deliberate use of formal and informal relationships as evidence advocacy channels.

As a heuristic device, the enquiry framework is subject to fallibility. Theoretical understanding of how framework components influenced each other over time was difficult to assess empirically. Whilst compelling data emerged on the political influence over PHN issues, collecting interview data over a relatively short time period created difficulty for developing a nuanced understanding of how politics influences the impact of advocacy for evidence use over time and across policy issues. A key limitation when collecting case study data was interviewees’ perceptions of evidence use. As divisions within the policy community centred on the evidence base for policy priorities, a number of interviewees struggled to separate the idea of advocacy for evidence use per se, from advocacy for consideration of their evidence.

### Advocacy for evidence use (AEU) framework

Insights from the case study produced three revisions to our ‘Role of Advocates for Using Evidence framework’ [16] incorporated into the AEU framework. First, a shift of focus from the role of advocates to advocacy processes and outcomes in three areas. Secondly, having politics as the alternative hypothesis led to recognition that political forces exert a more powerful contextual influence on advocacy than policy-relevant knowledge which, on reflection, is an outcome of deliberative policymaking processes. Thirdly, the overall advocacy goal, evidence-informed policymaking, moves from being an outcome to being integral to advocacy, portrayed in the centre of the AEU framework (Fig. 1). This captures how structures and systems promoting evidence-informed policymaking are enhanced by and enhance the impact of supportive meta-policy, sustained relationships and deliberative processes.

### The AEU framework and its propositions

Political theory can explain the influence of structurally powerful groups as coalitions of interests seeking common goals. Our study suggests that another political mechanism, advocacy, is a justifiable means for bringing about enhanced evidence use in policymaking. As all groups in the policy community held deep-level beliefs about the usefulness of evidence, a new frame for policymaking appears tenable. In this frame, the powerful exert their influence to change meta-policy towards structures and systems where the use of broadly based evidence is prioritised over other policy considerations. This proposition offers direction for the new approaches to PHN policymaking urged by Lang and Rayner [6], Swinburn et al. [21] and Rigby et al. [48].

**Fig. 2:**
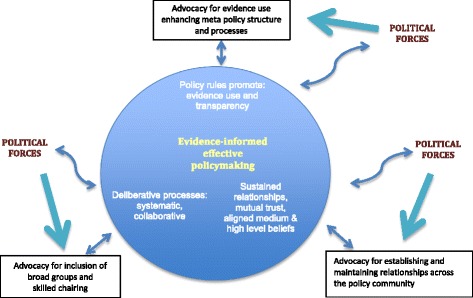
Advocacy for evidence use framework.

The AEU framework proposes policy structure and process change become advocacy goals for the PHN policy community. Opinion leaders across the PHN community have the capacity to use their social influence skills to persuade members of their networks and the government of the benefits of deliberative, transparent use of evidence. For the ‘idea’ of using evidence to become embedded in the hearts and minds of policy communities, opinion leaders themselves may first need to be persuaded of its value. Many of these influential individuals will reframe their evidence use advocacy from calls for government to develop policies directly informed by their evidence, to advocate for policy about policymaking in the broader public interest. The AEU framework (Fig. 2) proposes three concurrent advocacy targets for opinion leaders:

1. New governance models where meta-policy establishes and maintains government-led structures and processes that enable broadly based evidence to be considered. As PHN policy issues are inextricably linked with food industry activities, the relationship between values and evidence requires meta-policy rules that promote explicit and transparent processes. The exertion of asymmetrical power is avoided when policy processes recognise the value base of all contributors.
2. Deliberative policy processes that acknowledge the value of broadly based, systematically synthesised evidence. Individuals skilled in leading iterative processes will facilitate new collaborative approaches to developing understanding of and solutions to ‘wicked’ policy problems. This advocacy will address the role of government in ensuring skilled staff undertake evidence syntheses, which incorporate the values and interests of all contributors.
3. Establishment and maintenance of relationships across the policy community as the basis for effective advocacy. Opinion leaders persuaded of the benefits of on-going trusting relationships in advocacy for evidence use have the potential to assume a key role in facilitating relationships across the policy community. Their goal is to establish structures that enable relationships to exist over time and across issues. In addition, these leaders will be cognisant of the need for advocacy to be based on shared high and medium level beliefs.

The AEU framework (Fig. 2) illustrates the hypothesis that advocacy targeted to three inter-dependent areas, portrayed inside the circle, will bring about more evidence-informed effective policymaking. Opinion leaders engage in targeted advocacy (boxes) directed to three goals (inside circle) using iterative, responsive strategies (double-headed straight arrows). Wider political forces (outside the circle) exert influence in two ways, represented by wavy arrows capturing the less predictable influence of politics on each goal. These forces are distinct from deliberately harnessing politically influential advocacy in three areas, conveyed by the three straight one-way arrows between political forces and evidence use advocacy. The placement of political forces in the wider context also captures the influence of politics over the area that will be most malleable at a point in time. Interdependencies between each area capture the potentially synergistic actions that may be missed by focussing on an individual area.

## Conclusion

This research responded to growing pressure to better understand the PHN evidence–policymaking interface and strengthen PHN policymaking processes. Three channels were identified where advocacy will shift policymaking towards enhanced use of evidence: meta-policy, sustained relationships and deliberative policymaking processes. The principal theoretical contribution is adding understanding of the role of a politically mediated social interaction mechanism to conceptual models of evidence-informed policy. The AEU framework proposes that targeted advocacy for evidence use will build footbridges between decision makers and those who generate evidence. For policymakers and the PHN policy community the framework provides a theory-informed rationale for advocating for deliberate connections between science, society and politics. However, it also implies a central role for government in establishing evidence use promoting structures and systems. Another less obvious implication is the need for robust transparent systems for dialogue between all PHN actors where the influence of values is acknowledged. Finally, the framework provides the PHN policy community with urgently needed insights on the value of building advocacy constituencies for evidence use.

## Additional files

Additional file 1: Case study background. Description of the role of key players, the policy environment and key events in food marketing to New Zealand children, 2003–2014. (DOCX 166 kb)
Additional file 2: Interview guide. Question topic areas used in qualitative interviews with key members of the policy community. (DOCX 96 kb)

## Abbreviations

AEU: advocacy for evidence use
MoH: Ministry of Health
MPs: Members of Parliament
NGO: non-governmental organisation
PHN: public health nutrition
PPPs: public-private partnerships

## Footnotes

1 Dragon Dictate for Mac^©^, version 12116
