# Supplementary material for: Enhancing evidence use in public health nutrition policymaking: theoretical insights from a New Zealand case study
Source: Health Res Policy Syst. 2016 Nov 25;14:84. doi: 10.1186/s12961-016-0154-8 (PMC5124286; doi:10.1186/s12961-016-0154-8)
Supplement: Additional file 1: — Case study background. Description of the role of key players, the policy environment and key events in food marketing to New Zealand children, 2003–2014. (DOCX 166 kb) [file 12961_2016_154_MOESM1_ESM.docx]

Additional File 1

Case Study Background

Food Marketing to New Zealand Children

Introduction

Food marketing to children is a controversial policy issue in NZ. NZ policymakers unlike a number of their counterparts in the developed world have not instigated policies to promote healthy weight in children through the regulation of food marketing[[1-3](#_ENREF_1)].

Policymakers have maintained this position despite increasing concern by the global policymaking, health professional and research communities around the rising rates of childhood obesity[[4](#_ENREF_4)]. One in five (22%) NZ children are overweight and one in nine (11%) obese, increasing their risk of morbidity, disability and premature death [[5](#_ENREF_5)].

1. NZ policy on food marketing to children 2003-2014

New Zealand has had an industry self-regulatory framework for food marketing to children over this period[[6](#_ENREF_6)]. This policy approach persisted despite attempts by public health advocates to persuade policymakers to regulate. The framework endorsed a suite of self-regulatory codes administered by the industry-funded Advertising Standards Authority (ASA)[[7](#_ENREF_7)]. The Children's Code for Advertising Food 2010 was based on three principles:

- *“Food advertisements should not undermine the food and nutrition policies of Government, the Ministry of Health Food and Nutrition Guidelines nor the health and wellbeing of children.*
- *In interpreting the code, emphasis will be placed on compliance with both the principles and the spirit and intention of the code.*
- *Advertisements should comply with the laws of New Zealand and appropriate industry codes including the New Zealand Television Broadcasters code "Getting It Right for Children"*^[^[^6^](#_ENREF_6)^].^

Whilst the ASA code did not provide any restriction on television viewing times, the NZ Television Broadcasters’ Council Code (NZTBC) code disseminated by an association of television broadcasters ‘Think TV’ prohibited advertising in designated preschool television programming times. The NZTBC code also restricted advertising in school age children’s designated viewing times to ten minutes per hour, morning and afternoon, with restrictions ending no earlier than 5.30pm^[^[^8^](#_ENREF_8)^]^.

The ASA and NZTBC codes have been criticised on a number of grounds, including the following^[^[^2^](#_ENREF_2)^,^ [^3^](#_ENREF_3)^,^ [^9-11^](#_ENREF_9)^]^:

- School age children’s actual television watching times extend well beyond the 5.30pm cut off.
- Code restrictions apply only to television; other media are governed by principles only.
- Weak complaint mechanisms favour advertisers.
- The small number of complaints in recent years.
- The Code breaches the United Nations Rights of the Child: the right to restrict the freedom of expression in order to protect public health.
- Defining children as being under 14 years of age, not using the United Nations criteria of under 18 years.
- The Code does not address newer media to which children are increasingly exposed.
- Voluntary codes that rely on public complaints to achieve outcomes are a weak regulatory mechanism.

2. Stakeholders in NZ Food Marketing to Children Policy Community

Over the period 2003-2014, three discernable groups of NZ stakeholders were actively involved in food marketing to children issues. Each group had a unique view of the issue, draws on a particular evidence base and reflects a level of vested interest. The comparative summary in Table 1 below shows the nature of the food industry’s evidence base and key arguments that underpin current government policy. Public good arguments behind the public health groups and policymakers’ positions have not influenced successive governments. The activities of the influential food industry and the other groups resulted in an on-going self-regulatory policy in NZ. The activity of these groups is summarised below.

Table 1. Stakeholders in Food Marketing to Children in NZ (2003 – 2014)

|  | **Food Industry** | **Public Health** | **Policymakers** |
| --- | --- | --- | --- |
| **Key actors** | - Food Industry Coalitions - Individual food companies | - NGO coalitions - Individual academics - Individual NGOs | - MoH bureaucrats |
| **Major coalitions, organisations** | - Food Industry Group - Food and Grocery Council | - National Heart Foundation, - Agencies for Nutrition Action, - Obesity Action Coalition until March 2010, Chronic Disease Prevention Peak group (currently in abeyance) | - Not identifiable |
| **Key arguments** | - Self-regulation is effective, - Parental rights, - Autonomy, - Cost of regulation, - Marketing encourages brand switching not increased consumption | - Increasing child obesity, - Social environmental determinants, - Influence of marketing on children, - Cost benefit of regulatory policy | - Public Health legislation opportunity for government intervention on NCDs |
| **Key criticisms** | - Powerful vested interests have undue influence, - Evidence weak | - No causal evidence link between marketing and obesity | - Political interference, - Lack robust structures and processes |
| **Evidence base** | - Consumer marketing - Industry reports | - Nutritional science - Social psychology - Ethics, Human rights | - Nutritional science, - Political and economic risk |
| **Level of vested interest** | Very High | High | Medium – High |

Source: Author

2.1 Food Industry

Over these eleven years the food industry in NZ played an active role in shaping policy on food marketing to children. A summary of key initiatives follows.

In 2003, a defining event occurred when members of the food and advertising industries formed the Food Industry Group (FIG) in response to public debate and government interest in regulating food marketing to children. Key FIG members were representatives from the Association of New Zealand Advertisers, NZTBC, Communications Agencies Association of New Zealand and New Zealand Food & Grocery Council. During 2003 and 2004 FIG members undertook a range of activities to build relationships with government decision makers and selected academics^[^[^12^](#_ENREF_12)^]^. This initiative led to the FIG signing a voluntary agreement with the MoH in September 2004, known as the Food Industry Accord in which FIG committed:

“To do all that is possible to encourage all sectors of the food industry to create commercially successful products and services that will make a positive contribution to the health of New Zealanders”^[^[^13^](#_ENREF_13)^]^.

Between 2005 and 2008 the FIG members held fortnightly ‘Dialogue and Influence’ meetings with MoH officials^[^[^14^](#_ENREF_14)^]^. Industry representatives also used the 2006 Government Health Select Committee Inquiry into Obesity and Type 2 Diabetes to advocate for continued industry self-regulation. Their arguments for voluntary codes centred on the existing codes being socially responsible and effective, as the NZTBC’s submission illustrated:

“An effective, socially responsible fabric of rules and regulations exists across all advertising with television being at the forefront of offering a socially responsible approach in New Zealand (based around Broadcasting Standards Authority and Advertising Standards Authority codes)… this framework works well. The NZTBC has seen no information that the incidence of obesity would be reduced through greater regulation (s293, p4)”^[^[^15^](#_ENREF_15)^]^.

In critiquing the submission, it is noted: the statement that the incidence of obesity would not be reduced through regulation was not referenced. Other industry submissions to the Inquiry identified that industry framed their position as a matter of individual choice, autonomy and education:

“At the centre of the solution is the individual… Given the range and availability of food items on offer, the key is in giving people the knowledge and ability to make healthy choices. It comes down to teaching people the basic principles of how much they consume vs. how much they move…”

“We live in a democracy, not a dictatorship, and thus we cannot tell people that they cannot eat some foods but eat lots of others. We can only exhort. It is how well we exhort the consumption of healthy diets and living healthy lifestyles that will achieve the objectives of reducing obesity and the incidence of type 2 diabetes^[^[^15^](#_ENREF_15)^]^.

Furthermore, a frequently expressed argument was that more evidence was needed on appropriate obesity prevention strategies before policy action could be taken, as the FIG submission illustrates:

“Some people claim that even though we do not know enough about the causes of the problem, we must still act – as if panicking blindly will be more helpful than taking a moment to size up the situation. If we do not answer the questions above (about causes), then New Zealand runs the risk of attempting solutions which unnecessarily impact on all New Zealanders while being unlikely to make any significant impact on those who were obese (s157, p9)”^[^[^15^](#_ENREF_15)^]^.

Also relevant is the assertion by the FIG that their advocacy was evidence-based and highly influential:

“The submissions presented by industry have provided in-depth, evidence-based information that has made a major contribution to the debate”^[^[^16^](#_ENREF_16)^]^.

However, most of the evidence FIG cited was either unpublished reports or from minor journals^[^[^17^](#_ENREF_17)^]^. Nevertheless the Select Committee recommended to Government that self-regulation continue with the Food Industry Group and MoH jointly being given targets and timeframes to address the: “*advertising, marketing and promotion of healthier diets, especially to children*…”^[^[^18^](#_ENREF_18)^]^

In 2007 - 2008 a Government initiated review of NZ’s major and overarching piece of public health legislation the Health Act (1956), provided the food industry with another opportunity to advocate for self-regulation. In the draft revision of the Public Health Bill policymakers recommended a moderate level of government intervention for non-communicable diseases, including obesity[[19](#_ENREF_19)]. Despite policymakers proposing formal government - industry partnerships to oversee the increased regulation, food industry retained their position. A human rights argument was added to their previous case, as the submission by one large food company illustrated:

“Powers to regulate were unnecessary, unreasonable and conflicted with the Bill of Rights. To introduce overt state coercion in the food choices of citizens is going one step too far”^[^[^20^](#_ENREF_20)^]^.

Alongside and subsequent to these submissions to government bodies, the NZ food industry maintained a public profile around their public health promotion activities. These included involvement in community initiatives, working with NGOs notably the National Heart Foundation (NHF), maintaining relationships with the MoH and voluntary product reformulation^[^[^21^](#_ENREF_21)^]^. The 2011 - 2012 FIG annual report profiles a number of these activities. This report reveals a shift in the industry case for self-regulation, with the addition of cost of regulation and whole of society burden arguments:

“ There have been many proposals from lobby groups for taxes, bans and new labelling regulations that will add costs and inconvenience to all consumers not just those suffering from obesity. What’s more concerning is that many of these calls come with no supporting evidence that these impositions will actually solve the underlying problem.”^[^[^22^](#_ENREF_22)^]^

This 2011/ 2012 report also conveyed a high level of industry confidence in the relationship they have with the government of the day:

“It was refreshing that both New Zealand and overseas government bodies have embraced the place of industry self-regulation as part of the solution.”

Since 2003, the only industry initiated public consultation process was a 2010 revision of two ASA codes. Public submissions were invited to “assist” the review of the 2006 Food Code and Children’s Code. Thirty-five submissions were received including eight from organisations with industry associations^[^[^23^](#_ENREF_23)^]^. Of these eight, two of the three food companies and one of the three industry associations were interviewed for this research. As the submissions were not in the public domain and not under the jurisdiction of the Official Information Act 1982, it was not possible to record any additional observations about the use of evidence or evolution of the industry position on self-regulation.

2.2 Non-government public health advocates; NGOs and Academics

The second identifiable stakeholder group comprised NGOs, academic interest groups and professional associations who share a public health concern for childhood obesity.

Compared to the food industry coalition (FIG) NGOs involved a larger number of smaller organisations and individuals, most with lower levels of financial resource. In 2004 three nutrition NGO coalitions existed: the Obesity Action Coalition, Agencies for Nutrition Action and the Chronic Disease Prevention Peak group. These coalitions included the major nutrition NGO groups in NZ and had a high level of overlap in membership. The NHF and Diabetes NZ belonged to all three coalitions; the Cancer Society, Dietitians NZ and Te Hotu Manawa Maori (the indigenous branch of the NHF) belonged to two coalitions. Several of these coalitions had a chequered history. The Obesity Action Coalition funded by the MoH from 2003 until 2009, ceased to exist in 2010 when the funding policy of restricting advocacy activities was imposed[[24](#_ENREF_24)]. Peak group members struggled to reach agreement on a range of issues and the group went into abeyance. Agencies for Nutrition Action (ANA) remained as the only functioning nutrition NGO coalition. A politically active NGO, the Fight the Obesity Epidemic (FOE) group was the second casualty of government funding retrenchments, however unlike OAC, FOE is a charitable trust and has continued low-level activities since the 2009 funding cuts[[25](#_ENREF_25)]. As FOE’s top priority was prohibition of food advertising to children their large media advocacy role since 2000 made a notable contribution to the public profile of the issue.

Through partnerships with academics, these coalition groups and their individual members overtly positioned themselves as the ‘voice of scientific reason’. Their approach was to use scientific evidence to support their position: that obesity prevention needs to address the wider environmental and social determinants. International evidence and precedents were frequently cited as the basis for their case that government regulation is needed to influence the environmental determinants of obesity, as the NHF’s submission to the 2006 Health Select Committee inquiry on Obesity and Type 2 Diabetes illustrated:

“The government should consider obesity a normal response to an abnormal environment. Many of the determinants of obesity were structural and environmental and were outside the control of families/whanau and individuals. Therefore focusing interventions solely on educating people and trying to get them to ‘pull themselves up by their bootstraps … ignores all that we know about what determines health and well-being. Worse, it is an ineffective, naïve and futile approach that delays effective actions and widens … disparities”^[^[^26^](#_ENREF_26)^]^.

Other submissions by NGOs to the same Inquiry argued that evidence of ‘what works’ overseas should be applied to NZ:

“…international experience showed that voluntary codes and self-regulations did not bring about significant change for children”^[^[^27^](#_ENREF_27)^]^.

Although the NZ Medical Association is not a member of these NGO coalitions, they also argued the same pro-regulation position from a research-led evidence base. The association adopted the position held by the wider international medical community that:

“...some measures need to be taken to regulate the type of food advertising aimed at children”^[^[^28^](#_ENREF_28)^]^.

Typically, public health groups and community groups conducted their advocacy in the public domain through press releases, television and radio interviews. Whilst this media profile enabled their views to be shared with the New Zealand public, there was little coordination between groups and issues were usually short lived. Public health academics on the other hand only engaged with the media following the publication or presentation of a research paper of public interest or contributed to media debates upon request. NGO groups appeared to engage in media activities in response to topical political interest or to advance an issue of strategic importance to their organisation. Apart from the NZMA, other groups representing health professionals and especially Dietitians and Nutritionists had a very low media profile.

Both of the large nutrition NGOs - the NHF and ANA had developed evidence-based position statements and reports on food marketing to children^[^[^29^](#_ENREF_29)^,^ [^30^](#_ENREF_30)^]^. Although these documents contained recommendations for government policy, neither group actively disseminated their position. Early in its existence the Peak coalition group commissioned novel research with parents: the ‘2007 Survey of Public Opinions about Advertising Food to Children’[[31](#_ENREF_31)]. The Survey found that most parents and grandparents of children aged up to 13 years were very concerned about children being obese or overweight. They were also concerned about the role of food and drink advertising targeting children. Over 80 per cent were in favour of stopping advertising of unhealthy food and drinks to children^[^[^31^](#_ENREF_31)^]^. When released this research had a high media profile; however together with the Peak group both the report and the issue of expressed parental concern subsequently had a very low public profile.

2.3 Government policymakers

The third identifiable group of stakeholders was policy makers employed by government departments to provide policy advice to elected officials. Within government, influential individuals were more difficult to identify. In NZ’s political system, which is modelled on the Westminster system of government, employees of an apolitical government service are not able to make public statements. This right is afforded only to the Chief Executive or the Minister[[32](#_ENREF_32)]. Bureaucrats are directly answerable to politicians who can set clear expectations on the role of organisations outside government as the then Minister of Health, Hon Pete Hodgson illustrated in his address to a 2006 food industry conference:

“*Those of you sitting in this room, and the organisations you represent have the collective power to change New Zealand’s food environment - what is available to buy, how it is priced and how we purchase it. There is a strong sense of urgency to make these changes in light of the obesity epidemic we were now facing*”^[^[^33^](#_ENREF_33)^]^.

Despite these ‘signals’ from their Minister, health policy makers proposed a regulatory framework in the 2007 Public Health Bill which included food marketing. This draft legislation was released in the policy window following the presentation of the Obesity and Type 2 Diabetes Inquiry report and before the Government had produced their response^[^[^34^](#_ENREF_34)^]^. The proposed regulatory framework however had a short life following its introduction to Parliament in the first month of office of the next Minister of Health, the Hon David Cunliffe. The Health Select Committee (HSC) considering the Bill did not support regulatory policy. In their June 2008 report back to Parliament, this HSC recommended that voluntary codes continue, on the condition that targets and timeframes were met. To appease the majority of submitters who favoured regulation, the HSC recommended the Minister of Health be given powers to propose regulation if after two years there was no significant progress in achieving the target and timeframe objectives^[^[^35^](#_ENREF_35)^]^. The Government continued to signal support for self-regulatory policy in their response to the Obesity and Diabetes Inquiry report^[^[^36^](#_ENREF_36)^]^.

Throughout this period the minority political party, the Green Party remained strongly supportive of the policymakers’ and NGOs’ pro-regulatory position^[^[^37^](#_ENREF_37)^]^.

However, following a change of Government in November 2008 the Public Health Bill did not proceed in any form. The next Minister of Health, Hon Tony Ryall, expressed commitment to continuing to support industry self-regulation in his 2010 statement to advertising, communication and food industry representatives:

“Working with industry and food manufacturers constructively is an approach Government supports and we want to see more examples of the achievements in this area ”^[^[^38^](#_ENREF_38)^]^.

Because of this position by the 2008 – 2014 Government, NZ had continuing industry-controlled self-regulatory policy for food marketing to children.

Since the beginning of the National Party led Government’s term of office in 2008, the MoH pursued a focus on clinically measurable targets. These were originally set out in an overarching National Party manifesto ‘Better, Sooner, More Convenient’^[^[^39^](#_ENREF_39)^]^. During their first term the Government concentrated on reducing hospital waiting times and improving the quality and performance of the health system^[^[^40^](#_ENREF_40)^]^. In their second-term the Government started to address a small number of public health issues, for example rheumatic fever[[41](#_ENREF_41)]. However the only nutrition activity was the 2012 release of updated Food and Nutrition guidelines for children and older adults^[^[^42^](#_ENREF_42)^]^ and the July 2013 announcement of funding for up-skilling health professionals to deliver motivational food choice messages to mothers of young children^[^[^43^](#_ENREF_43)^]^.

**Conclusion**

Between 2004 and 2014, three broadly defined groups were active in the food marketing to children policy ‘space’ in NZ. The food industry undertook a number of initiatives that appeared to persuade the Government to discount the evidence of public health and NGO groups of a link between food marketing and rising rates of childhood obesity. The Government appeared to prioritise political agendas over the advice of bureaucrats who recommended regulation. This interaction occurred in the wider context of a growing body of international and national scientific evidence on the benefits of regulation, strong policy signals from the WHO, precedents established in other countries, and use of a precautionary approach on public health issues. The precautionary approach is argued to be appropriate for public health policy where there were threats of serious or irreversible damage, and lack of full scientific certainty is not a justifiable reason for inaction on measures which were known to be cost effective^[^[^44^](#_ENREF_44)^]^.

**References**

1. Bowers S, Signal L, Jenkin G: Does Current Industry Self-Regulation of Food Marketing to Children in New Zealand Protect Children from Exposure to Unhealthy Food Advertising ? In*.* Wellington: Cancer Society of New Zealand; 2012: 26.

2. Shaw C: (Non)regulation of marketing of unhealthy food to children in New Zealand. *New Zealand Medical Journal* 2009, 122(1288):76-86.

3. Wilson N, Signal L, Nicholls S, Thomson G: Marketing fat and sugar to children on New Zealand television. *Preventive Medicine* 2006, 42(2):96-101.

4. Bollars C, Boyland E, Breda J, Gapanenko K, Halford J, Klepp KI, Lobstein T, Xuereb G: Marketing of foods high in fat, salt and sugar to children: update 2012–2013. In*.* Edited by World Health Organization Regional Office for Europe. Copenhagen Ø, Denmark: WHO; 2013: 44.

5. Ministry of Health: Annual Update of Key Results 2014/15: New Zealand Health Survey. In*.* Wellington: Ministry of Health; 2015: 67.

6. New Zealand Advertising Standards Authority Children's Code For Advertising Food [<http://www.asa.co.nz/code_children_food.php>]

7. New Zealand Advertising Standards Authority Homepage [<http://www.asa.co.nz/>]

8. Getting it Right for Children [<http://www.thinktv.co.nz/thinktv/standards-and-regulations/children-an-tv/>]

9. Hoek J, King B: Food advertising and self-regulation: A view from the trenches. *Australian and New Zealand Journal of Public Health* 2008, 32(3):261-265.

10. Jenkin G, Signal L, Thomson G: Nutrition policy in whose interests? A New Zealand case study. *Public Health Nutrition* 2012, 15(08):1483-1488.

11. Thornley L, Signal L, Thomson G: Does industry regulation of food advertising protect child rights? *Critical Public Health* 2010, 20(1):25 - 33.

12. Irwin J: The Food Industry Group(FIG) 2nd Annual Report To the Minister of Health. In*.*: The Food Industry Group; 2006: 1-32.

13. N.Z. Food Industry Accord, The Health of Our Nation [<http://www.nztbc.co.nz/images/food_ind_accord.pdf>]

14. Status Report Document, Rationale for FIG Success [fig.org.nz/Resources/Library/Documents/.../Rationale_for_FIG_Success.doc]

15. White J: The Health Select Committee Inquiry into Obesity and Type Two Diabetes in New Zealand: An initial analysis of submissions. In*.* Wellington: Fight the Obesity Epidemic New Zealand Incorporated; 2007: 81.

16. New Zealand Food Industry Group Inc [<http://www.fig.org.nz/>]

17. Jenkin G, Signal L, Thomson G: Framing obesity: the framing contest between industry and public health at the New zealand inquiry into obesity. *Obes Rev* 2011, 12(12):1022 - 1030.

18. Kedgley S: Inquiry into Obesity and Type 2 Diabetes in New Zealand Report of the Health Committee. In*.*: NZ House of Representatives 2007: 49.

19. Ministry of Health: Public Health Bill. In*.* Edited by Health Mo. Wellington: New Zealand Government 2007.

20. Tait M: McDonalds argues against health bill regulation. In: *New Zealand Herald.* Auckland: Fairfax Media; 2008.

21. Food Industry Group: Capturing the Change,Helping to Address Obesity Issues in New Zealand A Snapshot of Action within the Food Industry Group @ December 2010. In*.* Auckland NZ: The Food Industry Group; 2010.

22. Irwin J: The Annual Report on the activities of the Food Industry Group for the year ended 31 March 2012. In*.* Auckland, NZ: Food Industry Group; 2012.

23. Advertising Standards Authority: Final Report on the Review of the Code for Advertising to Children and the Code for Advertising of Food. In*.* Wellington, NZ: Advertising Standards Authority; 2010: 26.

24. Ministry of Health: Principles to be observed by NGOs contracting with the Ministry of Health draft discussion document. In*.* Wellington, NZ: MoH; 2005: 3.

25. About FOE [<http://foe.org.nz/about-foe/>]

26. Obesity and Type 2 Diabetes Inquiry A submission prepared by the National Heart Foundation of New Zealand [<http://www.heartfoundation.org.nz/files/Submission_210406c.pdf>]

27. Oliver P: Tough food controls in anti-obesity bill. In: *New Zealand Herald.* Auckland; 2008.

28. NZ Medical Association submission to the Inquiry into Obesity and Type 2 Diabetes. In: *Health Select Committee.* Wellington: New Zealand Medical Association; 2006.

29. Advertising Food to Children Position Statement [[http://www.heartfoundation.org.nz/uploads/Advertising to children_Position Statement_Sept 2011_revised%281%29.pdf](http://www.heartfoundation.org.nz/uploads/Advertising%20to%20children_Position%20Statement_Sept%202011_revised%281%29.pdf)]

30. Scragg R, Quigely R, Taylor R: Does watching TV contribute to increased body weight and obesity in children ? In*.* Wellington: Agencies for Nutrition Action 2006.

31. Phoenix Research: Survey of public opinions about advertising food to children: Understanding attitudes in New Zealand. In*.* Auckland: Peak Group 2007.

32. Palmer GWR, Palmer M: Bridled power: New Zealand's constitution and government. Auckland [N.Z.]: Oxford University Press; 2004.

33. Hodson P: Address to food industry group seminar. In: *Food Industry Group Seminar.* Auckland 2006.

34. Ministry of Health: Marketing of Food to Children: Background Information and Policy Options. In*.* Wellington: Ministry of Health 2007.

35. Health Committee: Public Health Bill, Government Bill As reported from the Health Committee. In*.* Wellington: House of Representatives; 2008.

36. Ministry of Health: Government Response to the Inquiry into Obesity and Type 2 Diabetes presented to the House of Representatives in accordance with Standing Order 253 (J.1). In*.*: New Zealand Parliament; 2007.

37. Ad industry behaving like King Canute [<http://www.greens.org.nz/pressreleases/ad-industry-behaving-king-canute>]

38. Ryall T: Working with industry and food manufacturers constructively In: *Meeting with Communication and Advertsing Industry representatives and food industry CEOs.* 2010.

39. Ryall T: Better Sooner More Convenient. In*.* Wellington NZ: National Party; 2007.

40. Ryall T: The Government's Health Agenda. In: *Dean's Winter Lecture Series.* Auckland University of Technology: Beehive.govt.nz; 2009.

41. Budget 2013: Additional $21.3m to fight rheumatic fever [<http://www.beehive.govt.nz/release/budget-2013-additional-213m-fight-rheumatic-fever>]

42. Ministry of Health: Food and Nutrition Guidelines In*.* Edited by Moher D. Wellington, NZ: MoH; 2012.

43. Ryall T, J G: $2.3M to help mums and families make good food choices for their children. In*.* Wellington, NZ: NZParliament; 2013: 1.

44. Moodie R, Stuckler D, Monteiro C, Sheron N, Neal B, Thamarangsi T, Lincoln P, Casswell S: Profits and pandemics: prevention of harmful effects of tobacco, alcohol, and ultra-processed food and drink industries. *Lancet* 2013, 381(9867):670-679.
