# Supplementary material for: Enhancing evidence use in public health nutrition policymaking: theoretical insights from a New Zealand case study
Source: Health Res Policy Syst. 2016 Nov 25;14:84. doi: 10.1186/s12961-016-0154-8 (PMC5124286; doi:10.1186/s12961-016-0154-8)
Supplement: Additional file 2: — Interview guide. Question topic areas used in qualitative interviews with key members of the policy community. (DOCX 96 kb) [file 12961_2016_154_MOESM2_ESM.docx]

# Additional File 2

# **Case Study Interview Guide**

A: Policy Context

In your work as a ... what has been your involvement in advocating for public health nutrition policy change.

Probe involvement with advertising food on television to children issue.

B: Questions

1. The first aspect is the ‘rules’ for policymaking processes. Both the formal and informal processes that allow evidence to be considered or prevent it being considered.

|  | **Questions** |
| --- | --- |
| **1.1** | When you were in meetings where the actual [food advertising to children] policy issue or frame was being debated, how was the evidence underpinning each view of the issue considered? |
| **1.2** | How was evidence talked into becoming the basis for the dominant policy frame? |
| **1.3** | *If* changing the policymaking rules to give consideration of relevant evidence a higher priority when the policy frame is being negotiated – what sort of advocacy is needed to bring this about? |

**2.** Next aspect is the relationships between those who generate research evidence and those who use it. The evidence use literature suggests that where trust is built in relationships people more easily understand each other's views and use evidence to inform their thinking.

| **2.1** | How have your relationships with other members of the food and nutrition policy community continued across policy issues? |
| --- | --- |
| **2.2** | What advocacy has there been for on-going relationships between the policy and research communities?  Whose initiative to maintain these relationships? |
| **2.3** | *If* on-going relationships between researchers and the policy community helped evidence to be considered when the policy frame was being negotiated, what is needed to sustain these relationships? |

3. Last aspect is the deliberate consideration of a range of evidence informed views on a policy issue.

| **3.1** | What advocacy took place to consider a range of views on the actual policy problem?  What happened when evidence was used to reframe a policy issue? |
| --- | --- |
| **3.2** | What has the NZ Food and Nutrition policy community learnt from the development of self-regulatory policy on Food Advertising to Children? |
| **3.3** | *If* structured, transparent processes to consider a range of evidence informed views would enhance the consideration of evidence, then what is needed to make this happen? |

4. Final topic is the influence of politics.

| **4.1** | How did individuals/groups with high political power use evidence to support their framing of the policy issue? |
| --- | --- |
| **4.2** | How did individuals/groups with low political power use evidence to support their framing of the policy issue? |
| **4.3** | *If* politics dominates the development of policy frames, how can evidence be used more effectively? |

C: Additional observations

Any other experiences you have had advocating for evidence to be used in a policy making process.
